# Mental health outcomes in dementia caregivers: a systematic review of yoga-based interventions

**DOI:** 10.1590/1980-5764-DN-2024-0151

**Published:** 2025-01-27

**Authors:** Paula Pillar Pinto, Andrea Camaz Deslandes, Helena Moraes

**Affiliations:** 1 Universidade Federal do Rio de Janeiro, Instituto de Psiquiatria, Rio de Janeiro RJ, Brazil.

**Keywords:** Complementary Therapies, Quality of Life, Alzheimer Disease, Yoga, Caregivers, Terapias Complementares, Qualidade de Vida, Doença de Alzheimer, Yoga, Cuidadores

## Abstract

Mind-body interventions have been explored to enhance the psychological well-being of dementia caregivers; however, the specific effects of yoga practice remain underexamined.

**Objective:**

The aim of this study was to investigate the benefits of yoga on quality of life (QoL), life satisfaction, psychological well-being, attention, self-compassion, perceived stress, anxiety, depression, and caregiver burden for dementia caregivers.

**Methods:**

A comprehensive search was conducted on September 11, 2024, in databases including SciELO, PubMed, BVSalud, Web of Science, Embase, and PsycINFO, focusing on the effects of yoga for informal dementia caregivers compared to passive or active control groups through randomized and non-randomized trials. An initial review reduced 284 to 180 unique records; 172 studies were excluded, leaving 8 that met the inclusion criteria. Two authors independently selected and extracted data using a data extraction sheet based on the Cochrane Consumers and Communication Review Group. The Cochrane Risk of Bias tools (ROB2 and ROBINS-I) were employed to evaluate bias risk.

**Results:**

The review included three randomized and five non-randomized studies with a total of 161 participants, predominantly female (wives and daughters), with ages ranging from 39 to 76 years. Four studies utilized hatha yoga, while four employed non-traditional yoga. Significant improvements were observed in QoL, depression, stress, anxiety, and self-compassion, but not in caregiver burden. Furthermore, positive effects were more pronounced in face-to-face or online interventions than in recorded videos. On average, studies exhibited a moderate risk of bias.

**Conclusion:**

Yoga practice can serve as an effective intervention for enhancing the psychological aspects of dementia caregivers.

## RESUMO

Intervenções mente-corpo têm sido amplamente investigadas para melhorar aspectos psicológicos dos cuidadores de pessoas com demência. No entanto, a prática de yoga carece de análise aprofundada.

**Objetivo:**

Investigar os benefícios da yoga na qualidade de vida (QV), satisfação com a vida, bem-estar psicológico, atenção, autocompaixão, estresse percebido, ansiedade, depressão e sobrecarga do cuidador.

**Métodos:**

Foi realizada uma pesquisa em bancos de dados como SciELO, PubMed, Bvsalud, Web of Science, Embase e PsycINFO, focando no efeito da yoga para cuidadores informais de demência em comparação a grupos passivos ou ativos, através de estudos randomizados e não randomizados. A última busca foi rodada dia 11/09/2024. A revisão inicial reduziu 284 registros para 180, 172 foram excluídos, restando 8 que atenderam aos critérios de inclusão. Dois autores, independentemente, selecionaram e extraíram dados dos estudos incluídos usando uma planilha baseada no Cochrane Consumers and Communication Review Group. O risco de viés foi avaliado com as ferramentas Cochrane Risk of Bias (ROB2 e ROBINS-I).

**Resultados:**

Foram incluídos três estudos randomizados e cinco não randomizados, com 161 participantes, cuja idade média variou de 39 a 76 anos, a maioria do sexo feminino (esposas e filhas). Quatro estudos utilizaram hatha yoga e quatro, yoga não tradicional. Resultados significativos foram observados em QV, depressão, estresse, ansiedade e autocompaixão, mas não na sobrecarga do cuidador. Intervenções presenciais ou online mostraram resultados mais expressivos que vídeos gravados. O risco de viés foi moderado.

**Conclusão:**

A prática de yoga pode ser uma intervenção eficaz para melhorar aspectos psicológicos dos cuidadores.

## INTRODUCTION

The aging of the population brings with it a series of conditions that have a significant impact on public health and the homes of those who live with older people. The demographic landscape forecasts a substantial rise in the elderly population. The World Health Organization (WHO) projects that by 2030, one in six individuals globally will be 60 years or older. By 2050, the world’s population of people aged 60 years and older is expected to double, reaching 2.1 billion^1^. This demographic shift is accompanied by an upsurge in dementia cases among older adults. Over 10 million new cases of dementia are reported each year worldwide, implying one new case every 3.2 s^2^.

The characteristics of dementia necessitate continuous care and support for affected individuals. Providing care for people with dementia (PwD) often extends beyond the duration of caregiving for other elderly individuals, with nearly one-third of caregivers assisting someone living with dementia for five years or longer^3^. Caregivers providing informal support to PwD face a heightened risk of developing diverse health issues that significantly affect their quality of life (QoL) and overall well-being. Caring for individuals with dementia often involves assisting with daily tasks such as bathing, addressing incontinence, administering medications, facilitating mobility and dressing, and the management of bedsores^3^. Moreover, there is a daily psychological burden due to neuropsychiatric disorders and the memory loss of the patients.

Some models of factors leading to caregiver stress have been proposed. In the Poulshock and Deimling^4^ model, dementia leads to a caregiving burden that can manifest as strain in various ways, potentially exacerbated by factors such as behavioral disturbances or the caregiver’s physical and psychological ill-health. Pearlin and colleagues’^5^ model of caregiver stress outlined four key areas that contribute to caregiver stress: the background context (e.g., level of support and the impact of other life events), primary stressors related to the illness (such as the patient’s level of need and behavioral and psychological problems associated with dementia), secondary role strains (such as family conflict and disruptions to social life), and intrapsychic strains (including the caregiver’s personality, sense of competence, and feelings of role captivity). In Campbell et al.^6^ review of the model, the strongest predictors of caregiver burden were a sense of “role captivity” (carer feelings of being “trapped” in their role), caregiver overload (e.g., fatigue and burnout), adverse life events outside of the caregiving role, and the quality of the caregiver-patient relationship.

Interventions involving psychotherapy and psychoeducation strategies have been used to reduce the burden and improve the QoL in this population^7^. More recently, holistic approaches have also been recognized as effective for caregivers. A previous meta-analysis observed that meditation provides a small to moderate benefit for informal caregivers^8^. Another systematic review considered mindfulness-based interventions (MBI), and findings also evidenced post-treatment effects ranging from medium to large for caregiver stress and burden and large effects for QoL^9^. Aside from these practices, yoga is also regarded as one of the most common complementary health approaches in many countries^10^. Specifically, yoga is considered a practice that integrates behavioral, physical, mental, and spiritual aspects through movements, awareness, postures, breathing, meditation, chanting, and relaxation. As a result, its effects differ from those of physical exercise and MBIs^11^. Over the past decade, yoga has attracted increasing attention from the medical scientific community, driven by a greater recognition and understanding of mind-body connections in various medical disorders^12^.

However, this ancient Indian practice has undergone changes and developments. The classic yoga practices considered the eight limbs of yoga, which included external and internal ethical conduct (*yama* and *niyama*), physical postures (*asana*), breath control (*pranayama*), withdrawal of the senses (*pratyahara*), concentration (*dharana*), meditation (*dhyana*), and oneness with the object of ego dissolution (*samadhi*)^13,14^. In addition, *asana, pranayama,* relaxation, and meditation are included. Consequently, regular yoga practice has been associated with improvements in mental health, such as burden, stress, anxiety, depression, mindfulness, resilience, affect, happiness, well-being, satisfaction with life, self-compassion, and social relationships^15,16,17,18^.

Despite this evidence, these studies have shown different protocols and findings, so it needs systematic reviews to summarize the specific effects of yoga in PwD caregivers. A previous systematic review observed stress reduction with a positive impact on caregiver mental health^19^. However, the authors have also included mindfulness and meditation intervention studies. This discrepancy highlights the need for a comprehensive examination of the impacts of yoga interventions in the context of dementia caregiving. A previous integrative review has also concluded that yoga may be useful in reducing stress, depression, and anxiety while increasing the QoL indicators, vitality indicators, self-compassion scores, and mindfulness attention^20^.

Therefore, the present review aimed to investigate the effect of yoga on QoL and mental health aspects of informal caregivers of PwD.

## METHODS

This systematic review was conducted following the Preferred Reporting Items for Systematic Reviews and Meta-Analysis (PRISMA) 2020 guidelines, including both the checklist and expanded checklist^21^. The instruments used have been included in the Supplementary Material (available at https://www.demneuropsy.com.br/wp-content/uploads/2024/11/DN-2024.0151-Supplementary-Material.docx).

### Eligibility criteria

Types of participants: adults or older adults serving as informal caregivers of PwD.

#### Types of intervention

Interventions included all styles of yoga. Yoga, as practiced in India, is largely rooted in *Patanjali* or *Hatha* yoga, an umbrella term for the various styles that have emerged since yoga’s adoption in the West^22^. Therefore, yoga is defined traditionally as a combination of *ásanas*, *pranayama*, and *meditation*. Studies with multimodal interventions that combine yoga plus another active treatment (e.g., cognitive behavior therapy) were included if most of the intervention consisted of yoga. Mindfulness-based stress reduction interventions, transcendental meditation, *Vipassana*, or other meditation-only studies were not included.

#### Types of comparators

Comparators include passive or active control groups, waiting lists, aerobic exercise programs, health education programs, or other interventions.

#### Types of outcomes

The main outcome was the QoL of caregivers measured through validated instruments administered at least twice during the study (pre- and post-intervention). Moreover, some secondary outcome measures were taken into consideration, such as life satisfaction, psychological well-being, attention, self-compassion, perceived stress, anxiety, depression, and caregiver burden. There were no restrictions on the instruments used to measure additional outcomes.

#### Types of studies

Randomized controlled trials (RCTs) and non-RCT pre–post-intervention studies investigating the mental health outcomes in informal dementia caregivers who underwent comprehensive yoga-based interventions. No language, country, publication date, or publication status restrictions were imposed.

Consequently, studies were excluded if they were not clinical trials, whether randomized or not; if they investigated a population other than informal caregivers; or if they did not use a comprehensive yoga practice as the primary intervention.

### Information sources

A systematic search was conducted across multiple electronic databases, including SciELO, PubMed, Bvsalud, Web of Science, Embase, and PsycINFO. The search strategy comprised relevant terms related to dementia, Alzheimer, caregiver, and yoga. A manual search of initially selected studies’ lists was performed to identify additional potentially eligible studies. Furthermore, the reference lists and citations of eligible studies were meticulously reviewed to ensure a comprehensive identification of relevant literature. No language restrictions were applied during the search process. Efforts were made to include studies in languages other than English, and foreign-language papers were translated to ensure a comprehensive examination of the available evidence. The final search was conducted on September 11, 2024.

### Search strategy

A systematic search was conducted using relevant terms related to the intervention, the population, and the condition. The search strategy encompassed Boolean operators, truncation, and MeSH terms where applicable. Words not described as MeSH terms but related to the PICO’s (patient, intervention, comparison, and outcome) strategy were also included. The reference lists and citations of eligible studies were meticulously reviewed to ensure a comprehensive identification of relevant literature. No language restrictions were imposed on the search, and efforts were made to retrieve and translate relevant non-English publications. No restrictions were applied to the publication date. Of the six databases used, filters were applied to only two of them. At PsycINFO the filter “academic journals” and at SciELO the filter “article.”

### Selection process

Two authors (P.P.P. and H.M.) independently conducted a rigorous review and assessment of articles identified through electronic searches. The screening process was performed in an unblinded and standardized manner, where each record and report retrieved underwent meticulous evaluation for adherence to the inclusion criteria.

Furthermore, following the elimination of duplicate entries, a dual-reviewer approach was adopted for the assessment of titles and abstracts to determine study eligibility. Both reviewers independently procured and thoroughly examined the complete texts of studies deemed potentially relevant, engaging in subsequent discussions to address and resolve any uncertainties regarding trial inclusion. In instances where discrepancies emerged, a third reviewer participated in resolving any disagreements, fostering consensus.

### Data collection process

We developed a data extraction sheet based on the Cochrane Consumers and Communication Review Group’s data extraction template. Two independent and unblinded reviewers meticulously performed data extraction from the selected studies. A characterization table was systematically completed to encompass various study characteristics, including general identification number (ID general), database, search date, keywords, filters, source, title, DOI, and publication type. To ensure precision and reliability, the collected information from each study underwent a thorough cross-checking process.

### Data items

#### Types of participants

Age, sex, scholarity, and kinship were recorded to delineate the demographic characteristics of the study population.

#### Types of interventions

Detailed information on the yoga-based interventions was collected, including the type, dose, duration, and frequency. The interventions were compared against waiting list conditions or other interventions, with a comprehensive assessment of their respective types, doses, durations, and frequencies.

#### Types of outcome measures

Various outcome measures were systematically documented, including changes in QoL scores, as well as secondary outcomes such as caregiver burden, depression, anxiety, perceived stress, and self-compassion, all assessed using validated scales.

Missing data or unclear information was informed as in Table 1.

**Table 1:** Characteristics of included studies.

Reference	Sample	Intervention		Outcome measures and instrument	Findings
		Duration and frequency	Type and description		
Araujo et al.^27^ Origin: Brazil RCT	Sample size:49 Mean age: G1: 54.9±9 and G2: 53.5±8 Gender: Female Scholarity: 4–13 y Kinship: Most of them daughter Diagnosis of patient: CDR 1 and 2	8 weeks 1x/wk. G1 60 min. G2 30 min. G1: online G2: online + yoga videos	G1: Psychoeducation + Hatha Yoga. G2: Psychoeducation program	QoL: CQOL—AD Burden: ZARIT Depression, anxiety, and stress: DASS-21	G1 and G2 ↑ physical health, G1 ↑ friends, G2 ↑ memory and money. ↓ with no difference between groups NS
Danucalov et al.^25^ Origin: Brazil RCT	Sample size: 46 Mean age: G1: 53.4±8 and G2: 55.55±8 Gender: ±90% female Scholarity: NI Kinship: NI Diagnosis of patient: AD	8 weeks 3x/wk. 75 min. In-person x1 and DVD x2	G1: Hatha yoga and compassion meditation program (YCMP) G2: Waitlist	QoL: WHOQOL-BREF Self-compassion: SCS	↑ for G1 and NS for G2 ↑ for G1 and NS for G2.
Danucalov et al.^26^ Origin: Brazil RCT	Sample size: 46 Mean age: G1: 53.4±8 and G2: 55.55±8 Gender: ±90%female Scholarity: NI Kinship: NI Diagnosis of patient: AD	8 weeks 3x/wk. 75 min. In-person x1 and DVD x2	G1: Hatha yoga and compassion meditation program (YCMP) G2: Wait list	Stress: LSSI Anxiety: BAI Depression: BDI	↓ for G1 and NS for G ↓ for G1 and NS for G2 ↓ for G1 and NS for G2.
Allende et al.^29^ Origin: USA	Sample size: 15 Mean age: 63.8±9 Gender: 71% female Scholarity: 12–18 Kinship: NI Diagnosis of patient: Mild AD	12 weeks 1x/wk 75 min. Online Homework exercises 15–20 min. 5x/wk	Teleyoga and Kirtan Kriya meditation.	QoL: SF-36 Burden: RMBPC Depression: BDI	↓ Fatigue NS for Burden ↓ Depression
Balasubramanian et al.^23^ Origin: USA	Sample size: 15 Mean age: 68.7±8 Gender: 70% female Scholarity “some college to post-graduate” Kinship: NI Diagnosis of patient: Dementia and AD	12 weeks 5x/wk 60 min. Via app.	Gentle yoga and yoga breathing (GYYB)	Burden: ZARIT	NS for Burden
Waelde et al.^30^ Origin: USA	Sample size: 15 Mean age: 39–69 years ± Gender: Female Scholarity: 6–16 years Kinship: Spouses and daughters Diagnosis of patient: Dementia	6x/wk 5 days of 90 min + 1 day of 3 h in person. Homework: 30 min daily via audiocassettes and manual for home practice	Inner resources protocol (hatha yoga and meditation)	Depression: CES-D Anxiety: STAI Burden: RMBPC	↓ Depression ↓ Anxiety NS for Burden
Parkman and Olausson^23^ Origin: USA	Sample size: 5 Mean age: 55–65 years Gender: Female Scholarity: NI Kinship: Spouses and daughters Diagnosis of patient: Dementia	8 weeks 3x/wk 60 min. Online x1 and recorded x2	Yin yoga	Burden: ZARIT Self-compassion: SCS	NS for Burden NS for Self-compassion
Kelechi et al.^28^ Origin: USA	Sample size: 16 Mean age: 55 or older Gender: 70% female Scholarity: some college to post-graduate Kinship: NI Diagnosis of patient: Dementia and AD	12 weeks 5x/wk 30 min. videos	Gentle yoga and yoga breathing	QoL: EQ-5D 3L Depression: CES-D-10 Depression and anxiety: PROMIS	NS for QoL NS for Depression NS for Depression and Anxiety

Abbreviations: RCT, randomized controlled trial; CDR, Clinical Dementia Rating; QoL, Quality of life; NI, Not informed; NS, Not significant; AD, Alzheimer’s disease.

### Study risk of bias assessment

**Figure 1:**
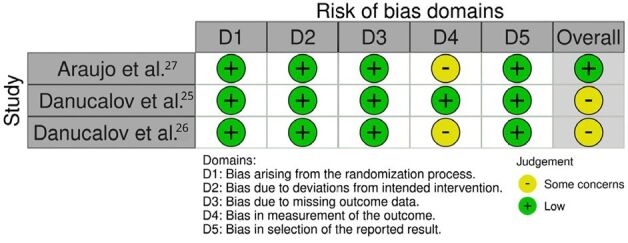
Risk of bias graph.

**Figure 2:**
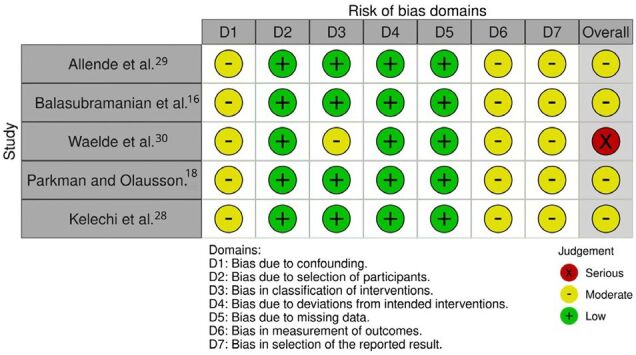
Risk of bias summary.

The Cochrane Risk of Bias (ROB2)^23^ for RCTs and Risk of Bias in Non-randomized Studies of Interventions (ROBINS-I)^24^ assessment tools served as the methodological frameworks for evaluating the quality and risk of bias in each included trial. Figures 1 and 2 illustrate the risk of bias assessment.

### Synthesis methods

Given the study’s nature and the absence of a meta-analysis, the synthesis primarily followed a systematic approach. Eligible studies underwent thorough evaluation, and intervention characteristics were systematically tabulated and compared against planned groups. Descriptive summaries, including tables and figures, were utilized to present key findings systematically and clearly. The Cochrane Risk of Bias tool (ROB2)^23^ for RCTs and ROBINS-I^24^ for non-randomized studies were employed to assess study quality and bias risk.

Results from individual studies were systematically compiled, and the structured synthesis provided a comprehensive interpretation of the collective evidence. Reporting bias was addressed by evaluating the risk associated with missing results, ensuring a thorough assessment of potential biases, despite the absence of a formal meta-analysis.

### Reporting bias assessment

A meticulous reporting bias assessment was undertaken. The Cochrane Risk of Bias tool (ROB2)^23^ for RCTs and ROBINS-I^24^ for non-randomized studies were instrumental in evaluating the quality and risk of bias of the studies.

To assess reporting bias, a thorough examination of trial registries, study protocols, and published literature was conducted. The reported outcomes within the included studies were compared against pre-specified outcomes in the study protocols, ensuring alignment with the planned research.

## RESULTS

### Study selection

**Figure 3:**
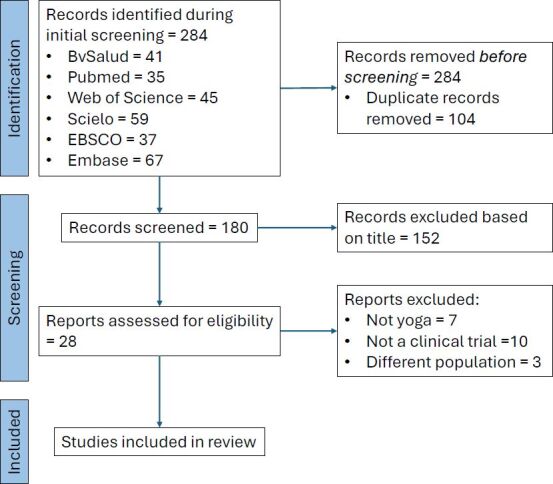
Prisma flow diagram.

The initial identification yielded a pool of 284 trials, from which duplicates were removed, resulting in the examination of 180 unique records for eligibility. In total, 152 studies were excluded based on the title because they were systematic reviews, qualitative studies, targeted a population other than informal caregivers of PwD, or addressed unrelated topics. Although 28 studies initially appeared to meet the inclusion criteria, a thorough methodological assessment revealed that 5 did not offer a complete yoga practice, which in this review is defined as including *āsanas*, *pranayamas*, and meditation. These five studies used only meditation. Additionally, two studies did not distinguish yoga from other forms of exercise in the reported outcomes. In total, 10 were not clinical trials, and three involved a different target population. As a result, this review included a total of eight studies. The PRISMA flow diagram (Figure 3) visually illustrates the thorough search strategy. Table 1 presents a summary of all relevant information regarding the studies included in this review.

### Study characteristics

Of the eight articles included in this review, three were RCTs^25,26,27^ conducted in Brazil, and five were non-randomized pre-post-intervention studies^16,18,28,29,30^ conducted in the United States, covering the publication period from 2004 to 2024. These collaborative efforts involved contributions from 37 distinct researchers and were published in journals related to complementary and alternative medicine (n=3), geriatrics and gerontology (n=3), nursing research (n=1), and clinical psychology (n=1).

All studies employed validated scales to measure their outcomes. Below is a list of the outcome types and the corresponding scales used, along with their respective references. To measure QoL, the studies utilized Alzheimer’s Disease Quality of Life Scale, caregiver version (CQOL-AD)^31^, World Health Organization Quality of Life questionnaire (WHOQOL-BREF)^32^, Short Form Health Survey-36 (SF-36)^33^, and The EuroQol (EQ-5D 3L)^34^. For caregiver burden, they have used the Burden Interview Scale (BI-Zarit)^35,36^, Revised Memory and Behavior Problems Checklist (RMBPC)^37^, and 12-item Zarit Burden Interview^38^. For perceived depression, Depression, Anxiety and Stress Scale (DASS-21)^39^, Beck Depression Inventory (BDI)^40^, BDI-II^41^, Center for Epidemiological Studies – Depression Scale (CES-D)^42^, and Patient Reported Outcomes Measurement Information System (PROMIS)^43^ were used. For anxiety, DASS-21, Beck Anxiety Inventory (BAI)^44^, and State-Trait Anxiety Inventory (STAI)^45^ were used. For perceived stress, PROMIS, DASS-21, and Lipp’s Stress Symptoms Inventory (LSSI)^46^ were used. Finally, for self-compassion, it has used the Self-Compassion Scale: SCS^47^.

### Risk of bias in studies

The risk of bias was assessed using different tools, as the review includes three RCTs and five non-randomized pre–post-intervention studies. Figure 1 illustrates the risk of bias assessment for RCT and Figure 2 for non-RCT.

Regarding the RCTs, two of the studies^25,27^ provided detailed information on random sequence generation. Given the nature of the intervention, neither participants nor care providers were blinded in any of the studies evaluated. Only one study^25^ explicitly reported blinding of outcome assessors. Due to the absence of blinding for outcome assessors, two RCTs were rated as having “some concerns” regarding the risk of bias.

For the single-arm studies^16,18,28,29,30^, all five were rated as having a moderate risk of bias due to confounding, primarily because the lack of a control group limits the ability to differentiate the effects of the intervention from potential confounding factors. Bias in the classification of interventions was minimal across the studies, with the exception of one study^30^, which did not report the *asana* protocol used and was therefore rated as having a moderate risk. Due to the absence of a control group, blinding of outcome assessors was not implemented. As a result, all non-randomized pre–post-intervention studies^16,18,28,29,30^ were rated as having a moderate risk in the measurement of outcomes domain. The absence of a control group to assess the relevance of reported outcomes also increases the potential for selective reporting of favorable or unfavorable results, leading to a moderate risk of reporting bias across these studies. In summary, four of the pre–post-intervention studies^16,18,28,29^ were classified as having a moderate overall risk of bias, while one^30^ was rated as serious.

### Results of individual studies

Characteristics of the sample, interventions, outcome assessment, and results are shown in Table 1. Of the eight included studies, three originated from Brazil^25,26,27^ and five from the United States^16,17,28,29,30^, being three RCTs^25,26,27^ and five non-RCTs^16,17,28,29,30^. A total of 161 participants were included in the studies; the sample size ranged from 5 to 49. Participants’ mean age ranged from 39 to 76 years. On average, 86% of the participants were female. When reported, the caregivers were predominantly wives and daughters of the PwD. The interventions varied, but all incorporated some form of technology (e.g., app, DVD, recordings, and online platforms) to enable home practice. All included core elements of yoga, such as *asanas*, breathing exercises, and meditative practices. On average, the interventions lasted 9.28 weeks, with a mean frequency of 4.2 sessions per week. Four studies used hatha yoga^25,26,27,30^, while the other four used other non-traditional yoga practices^16,18,28,29^. In total, four studies^25,27,28,29^ assessed QoL, and three^25,27,29^ have found significant improvement. In total, two studies^26,27,28^ assessed stress symptoms with significant results only for one^27^. Notably, five studies^26,27,28,29,30^ assessed depression severity with significant results for four studies^26,27,29,30^. A total of two^18,25^ studies assessed self-compassion with significant improvement for one^25^ and four^26,27,30^ studies that assessed anxiety, and three^26,27,30^ with significant results. It is also important to highlight that four^16,18,28,30^ of the eight studies were considered pilot studies.

In conclusion, this systematic review aimed to investigate the effect of yoga practices on the mental health of caregivers of PwD. Our focus rested on utilizing scientifically rigorous studies such as RCTs and also non-RCTs, but all with interventions that meticulously adhered to the fundamental principles of yoga. Upon analysis, the findings collectively indicate a potential positive impact of yoga on the mental health outcomes of caregivers, notwithstanding methodological variations among the included studies.

Yoga has emerged as a complementary therapeutic modality, offering potential benefits in the treatment of certain mental disorders alongside conventional interventions^48^. Additionally, its spiritual origins focused on self-realization suggest promising avenues for addressing psychiatric symptoms, potentially mediated by neurobiological mechanisms^49^. Despite this promise, the field of yoga therapy lacks standardized protocols for managing conditions such as anxiety, depression, and other mental issues^50^. It is noteworthy that while physical exercise has established efficacy in promoting mental well-being^51^, the physical aspects of yoga practices exhibit both similarities and notable distinctions^11^. Key differentiators include the emphasis on breath regulation, mindfulness, and posture maintenance within yoga sessions. Comparable to exercise, yoga offers advantages such as cost-effectiveness, non-invasiveness, minimal risk of adverse effects, and does not require medical supervision, while enhancing physical fitness. Thus, healthcare providers might consider integrating yoga into comprehensive patient care strategies^48^. Furthermore, when administered judiciously according to the personal belief systems of individual patients, these modalities can emerge as potent adjuncts to contemporary therapeutic approaches, potentially constituting the primary intervention in certain conditions^49^ or resonating with individuals averse to pharmacotherapy^11^. These outcomes have implications for the mental well-being of caregivers; however, it is imperative to delineate and analyze the precise outcomes observed within the context of this current review.

Regarding QoL, the present review observed an increase in total scores and specific domains after yoga practices. However, this improvement was more pronounced in a 75-min in-person session compared to the passive group^25^. In contrast, a 30-min online session showed no significant difference compared to the active group^27^. It is noteworthy that longer and in-person interventions tend to be more effective, although results may be influenced by the intervention in the comparison group. For instance, in Araújo’s study, the physical health domain of QoL increased in both the intervention (psychoeducation and yoga) and control (psychoeducation) groups. This could be attributed to the implemented psychoeducation program providing strategies to reduce overload and enhance QoL. Moreover, yoga practice promotes physical health and self-care. A non-controlled study has also observed QoL improvement, but only for fatigue items from QoL scale. It is important to highlight that these three studies have used different instruments to assess QoL, which can be influenced in the results^29^. A previous systematic review^52^ evaluated yoga’s effects on physical function and health-related QoL in older adults, revealing improvements in fitness aspects such as strength, balance, flexibility, and mental well-being.

On the other hand, anxiety, stress, and depression outcomes varied among studies. In the clinical trial that used a 30-min recorded video online intervention, no significant post-intervention effects were observed^27^. A non-significant difference was also observed in two other non-RCTs that used yoga videos^18,28^. Conversely, significant improvement across all mentioned dimensions was noted in a study conducting weekly 75-min in-person yoga sessions plus DVD^26^ and in-person intervention^30^, as well as for live online interventions^29^. Yoga, with its holistic approach, combining physical exertion with mindful breathing and energy regulation, has demonstrated various health benefits. A systematic review^53^ concluded that yoga influences cardiac autonomic regulation, resulting in increased heart rate variability and vagal predominance during sessions, potentially enhancing autonomic regulation and emotional well-being. Furthermore, previous reviews^54,55^ suggest yoga’s efficacy in significantly reducing depressive symptoms in elderly participants, and yoga also appears to be a promising modality for stress management. Moreover, the sense of belonging may exert influence on these outcomes. Defined as “a subjective feeling of value and respect derived from a reciprocal relationship to an external referent that is built on a foundation of shared experiences, beliefs or personal characteristics,”^56^ this sense intensifies when experienced in face-to-face interactions, fostering a deeper bond. In an online intervention, it is necessary to create and maintain a sense of community with personalized recognition on the part of the tutors, trying to ensure that the individuality of those being assisted is recognized instead of them being treated as mere numbers^57^. This observation is echoed in Araujo’s study, which noted challenges in observing and correcting students’ postures during yoga sessions. Consequently, conducting yoga sessions remotely can inhibit practitioners from being perceived and forming a meaningful connection with instructors, thereby limiting the potential positive impact on their well-being. Thus, it seems that these positive changes can be observed in in-person and online interventions, but not in recorded video interventions.

Other positive results in the selected studies considered as mental health outcomes were also investigated: self-compassion and burden. A significant improvement was observed for self-compassion in one study^25^, but non-significant for one pilot study^18^. On the other hand, burden was assessed in the other five studies^16,18,27,29,30^, but only one observed a significant reduction for the intervention group as well as the control group^27^. The search for quality articles utilizing yoga as a complementary integrative therapy for dementia caregivers has revealed a significant gap in such interventions for this population. Several barriers contribute to this difficulty, including transportation challenges for caregivers, privacy loss for online practice at home, and achieving a sufficient sample size for a randomized controlled study. Moreover, included studies reported that daily demands, time constraints, and patient’s health problems were questions that promoted missing results and experimental mortality.

The findings of the reviewed studies suggest a potential positive effect of yoga practice on mental health. One limitation of the present review is the small number of included studies, which can be explained by the inclusion of only studies that have used traditional yoga as a combination of *ásanas*, *pranayama*, and meditation in informal caregivers and pilot studies. Despite that, previous systematic reviews of mindfulness^58,59^ and an integrative review of yoga^20^ in this population have also included few studies. On the other hand, a previous systematic review included more studies, totaling around 13 yoga intervention trials; however, the authors also incorporated interventions that were exclusively or predominantly meditation-based^19^.

In addition, the analysis of the risk of bias classified the studies, on average, as moderate. In this sense, future research should prioritize high-quality studies that explicitly focus on the impact of yoga interventions on caregiver burden. This includes carefully designed interventions and robust methodologies that incorporate validated measures of caregiver burden. Furthermore, there is a need for comparative studies that systematically explore the differential effects of in-person and virtual yoga interventions. Understanding the nuances associated with session format, duration, and delivery mode can inform the development of interventions tailored to the preferences and constraints of caregivers.

In conclusion, the current evidence suggests a potential positive impact of yoga on QoL, stress, self-compassion, anxiety, and depression, but not for the burden of dementia caregivers, there are notable gaps and methodological challenges. Moreover, positive evidence is more expressive in face-to-face or online interventions than recorded videos. Addressing these gaps through targeted research efforts and methodological refinements will contribute to a better understanding of the role of yoga in supporting the well-being of informal caregivers in the context of dementia care.

## References

[1] World Health Organization (2021). Aging and health. https://www.who.int/news-room/fact-sheets/detail/ageing-and-health.

[2] Prince M, Wimo A, Guerchet M, Ali GC, Wu YT, Prina M (2015). World Alzheimer Report 2015. The global impact of dementia. An analysis of prevalence, incidence, cost and trends.

[3] Alzheimer’s Association (2020). Promoting caregiving across the full community: the role for public health strategists. https://www.alz.org/media/Documents/alzheimers-dementia-promoting-caregiving-across-full-community.pdf.

[4] Poulshock SW, Deimling GT (1984). Families caring for elders in residence: issues in the measurement of burden. J Gerontol.

[5] Pearlin LI, Mullan JT, Semple SJ, Skaff MM (1990). Caregiving and the stress process: an overview of concepts and their measures. Gerontologist.

[6] Campbell P, Wright J, Oyebode J, Job D, Crome P, Bentham P (2008). Determinants of burden in those who care for someone with dementia. Int J Geriatr Psychiatry.

[7] Jütten LH, Mark RE, Wicherts JM, Sitskoorn MM (2018). The effectiveness of psychosocial and behavioral interventions for informal dementia caregivers: meta-analyses and meta-regressions. J Alzheimers Dis.

[8] Dharmawardene M, Givens J, Wachholtz A, Makowski S, Tjia J (2016). A systematic review and metaanalysis of meditative interventions for informal caregivers and health professionals. BMJ Support Palliat Care.

[9] Shim M, Tilley JL, Im S, Price K, Gonzalez A (2021). A systematic review of mindfulness-based interventions for patients with mild cognitive impairment or dementia and caregivers. J Geriatr Psychiatry Neurol.

[10] Clarke TC, Black LI, Stussman BJ, Barnes PM, Nahin RL (2015). Trends in the use of complementary health approaches among adults: United States, 2002-2012. Natl Health Stat Report.

[11] Govindaraj R, Karmani S, Varambally S, Gangadhar BN (2016). Yoga and physical exercise – a review and comparison. Int Rev Psychiatry.

[12] Guddeti RR, Dang G, Williams MA, Alla VM (2019). Role of yoga in cardiac disease and rehabilitation. J Cardiopulm Rehabil Prev.

[13] Yatham P, Chintamaneni S, Stumbar S (2023). Lessons from India: a narrative review of integrating yoga within the US healthcare system. Cureus.

[14] Iyengar BKS (2020). Luz na vida: a jornada da ioga para a totalidade, a paz interior e a liberdade suprema.

[15] Csala B, Springinsfeld CM, Köteles F (2021). The relationship between yoga and spirituality: a systematic review of empirical research. Front Psychol.

[16] Balasubramanian S, Mueller M, Madisetti M, Hendrix K, Kelechi TJ (2023). Self-administered gentle yoga and yoga breathing intervention improves burden and stress biomarkers in caregivers of persons living with dementia. Int J Yoga Therap.

[17] Chhugani K, Metri K, Babu N, Nagendra HR (2018). Effects of integrated yoga intervention on psychopathologies and sleep quality among professional caregivers of older adults with alzheimer’s disease: a controlled pilot study. Adv Mind Body Med.

[18] Parkman S, Olausson J (2023). Effects of yin yoga on burden and self-compassion in caregivers of persons with dementia: a pilot study. J Gerontol Nurs.

[19] Martis CS, Chandrababu R, Ravishankar N, Bhandary RP, Mohammed CA, Tolson D (2023). The effectiveness of yoga therapy on caregivers of people living with dementia: a systematic review and meta-analysis of randomized controlled trials. Clin Epidemiol Glob Health.

[20] Parkman S, Olausson J (2023). Efficacy of yoga for caregivers of persons with dementia: an integrative review. Scand J Caring Sci.

[21] Page MJ, McKenzie JE, Bossuyt PM, Boutron I, Hoffmann TC, Mulrow CD (2021). The PRISMA 2020 statement: an updated guideline for reporting systematic reviews. BMJ.

[22] Jeter PE, Slutsky J, Singh N, Khalsa SBS (2015). Yoga as a therapeutic intervention: a bibliometric analysis of published research studies from 1967 to 2013. J Altern Complement Med.

[23] Sterne JAC, Savović J, Page MJ, Elbers RG, Blencowe NS, Boutron I (2019). RoB 2: a revised tool for assessing risk of bias in randomised trials. BMJ.

[24] Sterne JA, Hernán MA, Reeves BC, Savović J, Berkman ND, Viswanathan M (2016). ROBINS-I: a tool for assessing risk of bias in non-randomised studies of interventions. BMJ.

[25] Danucalov MA, Kozasa EH, Afonso RF, Galduroz JCF, Leite JR (2017). Yoga and compassion meditation program improve quality of life and self-compassion in family caregivers of Alzheimer’s disease patients: a randomized controlled trial. Geriatr Gerontol Int.

[26] Danucalov MAD, Kozasa EH, Ribas KT, Galduróz JCF, Garcia MC, Verreschi ITN (2013). A yoga and compassion meditation program reduces stress in familial caregivers of Alzheimer’s disease patients. Evid Based Complement Alternat Med.

[27] Araujo EL, Rodrigues MR, Kozasa EH, Lacerda SS (2023). Psychoeducation versus psychoeducation integrated with yoga for family caregivers of people with Alzheimer’s disease: a randomized clinical trial. Eur J Ageing.

[28] Kelechi TJ, Layne D, Mueller M, Madisetti M, Balasubramanian S (2024). Feasibility and preliminary impact of a web-based mind body intervention for older dementia caregivers. West J Nurs Res.

[29] Allende S, Mahoney L, Francisco JM, Fitz K, Keaney A, Parker-Bridges K (2024). Teleyoga for patients with Alzheimer’s disease and chronic musculoskeletal pain and their caregivers: a feasibility study. Glob Adv Integr Med Health.

[30] Waelde LC, Thompson L, Gallagher-Thompson D (2004). A pilot study of a yoga and meditation intervention for dementia caregiver stress. J Clin Psychol.

[31] Novelli MMPC (2006). Validação da escala de qualidade de vida (QdV-DA) para pacientes com doença de alzheimer e seus respectivos cuidadores/familiars [tese].

[32] Fleck MPA, Leal OF, Louzada S, Xavier M, Chachamovich E, Vieira G (1999). Desenvolvimento da versão em português do instrumento de avaliação de qualidade de vida da OMS (WHOQOL-100). Rev Bras Psiquiatr.

[33] Brazier JE, Harper R, Jones NM, O’Cathain A, Thomas KJ, Usherwood T (1992). Validating the SF-36 health survey questionnaire: new outcome measure for primary care. BMJ.

[34] EuroQol Group E (1990). EuroQol--a new facility for the measurement of health-related quality of life. Health Policy.

[35] Zarit SH, Zarit JM (1990). The memory and behavior problems checklist and the burden interview.

[36] Scazufca M (2002). Brazilian version of the Burden Interview scale for the assessment of burden of care in carers of people with mental illnesses. Rev Bras Psiquiatr.

[37] Allen RS, Burgio LD, Roth DL, Ragsdale R, Gerstle J, Bourgeois MS (2003). The revised memory and behavior problems checklist--nursing home: instrument development and measurement of burden among certified nursing assistants. Psychol Aging.

[38] Seng BK, Luo N, Ng WY, Lim J, Chionh HL, Goh J (2010). Validity and reliability of the zarit burden interview in assessing caregiving burden. Ann Acad Med Singap.

[39] Vignola RCB, Tucci AM (2014). Adaptation and validation of the depression, anxiety and stress scale (DASS) to Brazilian Portuguese. J Affect Disord.

[40] Beck AT, Steer RA (1993). Beck depression inventory manual.

[41] Smarr KL, Keefer AL (2011). Measures of depression and depressive symptoms: Beck Depression Inventory-II (BDI-II), Center for Epidemiologic Studies Depression Scale (CES-D), Geriatric Depression Scale (GDS), Hospital Anxiety and Depression Scale (HADS), and Patient Health Questionnaire-9 (PHQ-9). Arthritis Care Res (Hoboken).

[42] Radloff LS (1977). The CES-D scale: a self-report depression scale for research in the general population. Appl Psychol Meas.

[43] Pilkonis PA, Choi SW, Reise SP, Stover AM, Riley WT, Cella D (2011). Item banks for measuring emotional distress from the patient-reported outcomes measurement information system (PROMIS®): depression, anxiety, and anger. Assessment.

[44] Beck AT, Epstein G, Brown G, Steer RA (1988). An inventory for measuring clinical anxiety: psychometric properties. J Consult Clin Psychol.

[45] Spielberger CD, Gorsuch RL, Lushene R (1970). Manual for the state-trait anxiety inventory.

[46] Lipp ML (2000). Manual do inventário de sintomas de estresse para adultos de Lipp.

[47] Neff KD (2003). The development and validation of a scale to measure self-compassion. Self Identity.

[48] Panesar N, Shellharbour I (2011). Yoga and mental health. Australas Psychiatry.

[49] Varambally S, Gangadhar BN (2012). Yoga: a spiritual practice with therapeutic value in psychiatry. Asian J Psychiatr.

[50] Forbes B, Akhtar F, Douglass L (2011). Training issues in yoga therapy and mental health treatment. Int J Yoga Therap.

[51] Deslandes A, Moraes H, Ferreira C, Veiga H, Silveira H, Mouta R (2009). Exercise and mental health: many reasons to move. Neuropsychobiology.

[52] Sivaramakrishnan D, Fitzsimons C, Kelly P, Ludwig K, Mutrie N, Saunders DH (2019). The effects of yoga compared to active and inactive controls on physical function and health related quality of life in older adults- systematic review and meta-analysis of randomised controlled trials. Int J Behav Nutr Phys Act.

[53] Tyagi A, Cohen M (2016). Yoga and heart rate variability: a comprehensive review of the literature. Int J Yoga.

[54] Sharma M (2014). Yoga as an alternative and complementary approach for stress management: a systematic review. J Evid Based Complementary Altern Med.

[55] Wang YY, Chang HY, Lin CY (2014). Systematic review of yoga for depression and quality of sleep in the elderly. Hu Li Za Zhi.

[56] Mahar AL, Cobigo V, Stuart H (2013). Conceptualizing belonging. Disabil Rehabil.

[57] Peacock S, Cowan J, Irvine L, Williams J (2020). An exploration into the importance of a sense of belonging for online learners. International Review of Research in Open and Distributed Learning.

[58] Liu Z, Sun YY, Zhong BL (2018). Mindfulness-based stress reduction for family carers of people with dementia. Cochrane Database Syst Rev.

[59] Molero Jurado MDM, Pérez-Fuentes MDC, Barragán Martín AB, Soriano Sánchez JG, Oropesa Ruiz NF, Sisto M (2020). Mindfulness in family caregivers of persons with dementia: systematic review and meta-analysis. Healthcare (Basel).

